# A modified fluorimetric host cell reactivation assay to determine the repair capacity of primary keratinocytes, melanocytes and fibroblasts

**DOI:** 10.1186/1472-6750-10-46

**Published:** 2010-06-22

**Authors:** Katharina Burger, Katja Matt, Nicole Kieser, Daniel Gebhard, Jörg Bergemann

**Affiliations:** 1 Department of Biomedical Engineering, University of Applied Sciences, Anton-Günther-Strasse 51, 72488 Sigmaringen, Germany

## Abstract

**Background:**

The Host Cell Reactivation Assay (HCRA) is widely used to identify circumstances and substances affecting the repair capacity of cells, however, it is restricted by the transfection procedure used and the sensitivity of the detection method. Primary skin cells are particularly difficult to transfect, and therefore sensitive methods are needed to detect any variations due to the cell-type or inter-individual differences or changes induced by diverse substances.

A sensitive and repeatable method to detect the repair capacity of skin cells would be useful in two different aspects: On the one hand, to identify substances influencing the repair capacity in a positive manner (these substances could be promising ingredients for cosmetic products) and on the other hand, to exclude the negative effects of substances on the repair capacity (this could serve as one step further towards replacing or at least reducing animal testing).

**Results:**

In this paper, we present a rapid and sensitive assay to determine the repair capacity of primary keratinocytes, melanocytes and fibroblasts based on two wave-length Green Fluorescent Protein (GFP) and DsRed reporter technology in order to test different substances and their potential to influence the DNA repair capacity. For the detection of plasmid restoration, we used FACS technology, which, in comparison to luminometer technology, is highly sensitive and allows single cell based analysis.

The usefulness of this assay and studying the repair capacity is demonstrated by the evidence that DNA repair is repressed by Cyclosporin A in fibroblasts.

**Conclusions:**

The methodology described in this paper determines the DNA repair capacity in different types of human skin cells. The described transfection protocol is suitable for the transfection of melanocytes, keratinocytes and fibroblasts, reaching efficacies suitable for the detection of the restored plasmids by FACS technology. Therefore the repair capacity of different cell types can be compared with each other. The described assay is also highly flexible, and the activity of other repair mechanisms can be determined using modifications of this method.

## Background

The measurement of the ability to completely restore a (reporter-) gene was first described by Protic-Sablic in 1985 [1]. This rather complicated assay was modified by Athas et al. to measure the inter-individual variation in DNA repair capacity (DRC) in a large number of subjects [2]. In 2000, Roguev and Russev presented a modified HCRA using Green Fluorescent Protein and Yellow Fluorescent Protein as reporter genes, where luminescence was the marker for the restoration of the plasmid [3]. We have adapted the principle of this assay to FACS-technology. FACS-technology is highly sensitive in comparison to luminometer technology and allows single cell based analysis. It also detects slight differences between samples and facilitates working with cells that cannot be transfected easily. In contrast to the CAT assay, our modified assay determines the individual performance of every transfection and is therefore suitable to compare different experiments with each other. In the context of skin, the integrity of repair mechanisms plays a crucial role because the human skin acts as an external barrier and is subject to a constant barrage of DNA damaging substances such as exogenous chemical or physical agents and endogenous metabolites. The efficient removal of DNA lesions is one important mechanism to prevent malignant transformation and tumor progression [4-7]. The Host Cell Reactivation Assay is a promising tool in this context because it could serve to identify substances with positive effects on the repair capacity which could be used as active ingredients in cosmetics. The positive effect of folic acid on dermal fibroblast has already been proven using the Host Cell Reactivation Assay.

Another possible field of application lies within the confirmation/exclusion of negative influences on the repair capacity [8-11]. Such undesired effects of cosmetics or drugs could abet the development of malignancies. With respect to skin cells, cancer appears in the general population, not only as a result of exposure to sunlight, but also due to a reduction in DNA repair, as seen in XP patients [11,12]. For example, Cyclosporin A is associated with the development of skin malignancies [13-15] which could be caused by the inhibition of DNA repair.

An assay to exclude the negative influences of substances on the repair capacity of the major skin cells will make a contribution to the development of alternative methods to animal testing.

Although epidermal cells are considered to be the major target of UVB radiation as the vast majority of UVB is absorbed within the epidermis [16], there are only a few studies using dermal fibroblasts, probably because the used transfection methods are not suitable for the transfection of keratinocytes and melanocytes.

To overcome these limitations of transfection, we have developed a method to transfect main skin cell types, including primary skin cells which are difficult to transfect.

## Results and Discussion

In this study, a modification to an existing two wave length Host Cell Reactivation Assay [3] was developed based on flow cytometry. The reported observations demonstrate that it is possible to determine and compare the repair capacity of different primary human skin cells using the Host Cell Reactivation Assay.

### Isolation of Melanocytes, Keratinocytes and Fibroblasts from a single skin biopsy

Most of the described protocols for the isolation of primary human skin cells are only suitable for the isolation of either melanocytes or keratinocytes and fibroblasts. In order to assess a method which is suitable for comparing the repair capacities of different skin cell types, the cells were isolated from the same donor. All the cultures were obtained from skin biopsies obtained during various surgical procedures with the informed consent of the donor.

Our former studies, as well as studies done by others [8,9,17], have shown that the repair capacity is subject to extensive donor variation. Therefore, any comparison of the repair capacity of different skin cells requires that the individual cell types are isolated from the same biopsy. To achieve this requirement, the protocols had to be adapted - especially in the case of fibroblasts. According to our modified protocol, the tissue was processed in order to minimize microbial contamination and to remove subcutaneous elements as described earlier [18]. The removing of as much subcutaneous and fatty tissue as possible facilitates the separation of dermis and epidermis following Dispase treatment. Once dermis and epidermis were separated, they were treated with collagenase and trypsin respectively. After the treatment with trypsin, epidermal cells were collected by centrifugation, resuspended, quantified and plated. The collagenase treated dermal pieces were also plated. Normally dermal fibroblasts are isolated by plating undigested pieces of skin onto (covered) substrates [19]. This method works very well, but in our experience it takes up to three weeks until the cells can be passaged and separated from any contaminating keratinocytes. A digestion with collagenase is therefore favourable because it accelerates the outgrowth of the fibroblasts. The proliferation rate of keratinocytes is initially much higher than that of fibroblasts and therefore the required number of keratinocytes available for transfection is reached earlier. Furthermore, keratinocytes can only be kept in culture for four passages. Consequently, the keratinocytes have to be frozen until the required number of fibroblast is reached.

By implementing the digestion protocol for the isolation of fibroblasts described in this paper, the freezing and thawing of keratinocytes can be omitted. Additionally, since the availability of skin biopsies is limited, this method also insures the optimal utilization of the scarce resources.

### Transfection of Melanocytes, Keratinocytes and Fibroblasts

Transfection with a mixture of damaged and control plasmid is crucial for the Host Cell Reactivation Assay, however most primary skin cells are difficult to transfect. To compare the repair capacity of different cell types, a transfection procedure suitable for all cell types of interest was needed. First, we tried different established methods for the transfection. Using standard-procedures for calcium phosphate precipitation, we obtained transfection rates lower than 1% for the three cell types. Using the transfection method we described earlier [17], fibroblasts were transfected with very good results as transfection rates up to 40-60% were reached. This protocol is also suitable for melanocytes but, compared to fibroblasts, their proliferation rate is conspicuously lower and it is difficult to achieve the required number of cells. Keratinocytes cannot be transfected successfully using the electroporation method, although we tried different modifications.

**Figure 1:**
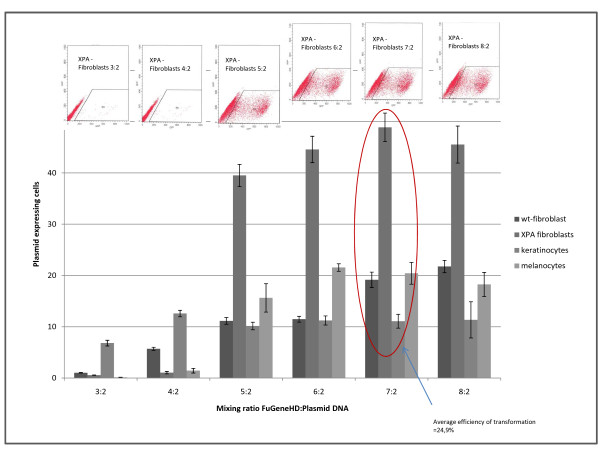
**Determination of the best mixing ratio of transfection reagent and DNA**. Primary fibroblasts, virus transformed XPA-fibroblasts (GM 000-Bürkle), keratinocytes and melanocytes were transfected with pEGFP-N1 using different mixing ratios of transfection reagent and DNA. Transfection efficiency was determined 24 h post transfection via FACS. The highest all over efficiency was found using a mixing ratio of 7:2.

For lipofection, we tested various reagents and decided to use FuGene HD transfection reagent (Roche). We tried different ratios of DNA and transfection complex according to the manufacturer's instructions in order to estimate the optimal mixing ratio. As figure 1 shows, the efficacies were varied, but the mixing ratio of 7:2 is suitable for the transfection of all three cell types and for the transfection of repair deficient fibroblasts which served as control. Using a 7:2 DNA:FuGene mix, we reached an average efficacy of 24.9%. In addition, we also conducted some experiments where we allowed complex formation to take place over different times periods without significant variations in efficacies (data not shown).

### Plasmids and UV irradiation

The expression of chloramphenicol acetyl transferase (CAT) as reporter can be detected in a variety of ways and has been the reporter of choice in reporter-vector systems for long time [20]. However, the notable disadvantage of these systems, however, is that the expressed reporter protein must be assayed via enzymatic reactions using whole cell extracts into which the reporter vector has been transfected [21].

Furthermore the quantification of DNA repair capacity via protein expression is disadvantageous because cells normally take up more than one plasmid, and therefore it is not possible to quantify the number of cells that are able to repair. With a view to the development of (skin-) malignancies, we also have to address the question whether a decrease in DNA repair capacity of a cell population rests on complete repair deficiency of single cells (while others remained unaffected) or on a partial decrease of DNA repair capacity in all cells. In our view single, repair deficient cells represent an even higher risk for the development of malignancies than a slight impairment of the DNA repair capacity in general.

In addition, the quantification via protein expression depends on the efficacy of the transfection, which never reaches 100%. Thus, it is impossible to conduct direct comparisons of the activity between different extracts [21]. Approaches to the solution of these problems are dual reporter systems as described by Thoms et al. [22] or in [21]. Since all of these systems are based on quantification via protein expression, they are nevertheless afflicted with the disadvantages named above.

The Discovery of fluorescent proteins opened up completely new vistas in the field of host cell reactivation and allowed to develop facilitated read out. In 2000, Roguev and Russev presented a modified HCRA using Green Fluorescent Protein and Yellow Fluorescent Protein as reporter genes and spectrofluorometric analysis (of all over fluorescence) to detect the restoration of the plasmid [3]. Green Fluorescent Protein (GFP) from luminescent jelly fish *Aequorea Victoria *has become an excellent marker for gene expression and protein localization in various biological systems [23]. Mutagenesis of the wild type gene yielded improved variants as well as colour variants such as Cyan and Yellow Fluorescent Proteins [24]. Unfortunately, the emission maxima of YFP and GFP excited by 488 nm laser light are in close proximity [3].Thereby, the fluorescence spectra of both proteins overlap in wide areas, so that they cannot be clearly distinguished using FACS-technology. However, the red fluorescent protein DsRed has spectral properties that are ideal for dual colour experiments with GFP [25], although it shows slow and complex kinetics of maturation, proceeding via a GFP like intermediate to the final red species [26]. Brooke and Glick introduced in 2002 a rapidly maturing variant of Dictosoma Red Fluorescent Protein. DsRed fluorescence is excided optimally at 358 nm but can also be excited by a 488 nm standard FACS laser [27]. Therefore we chose pEGFP-N1 (Clonetech; Cat. No. 6085-1 GenBank: U55762.1) and amodified DsRed plasmid for our FACS based host cell reactivation assay.

The main targets of the nucleotide excision repair are cyclobutan pyrimidin dimers and 6-4 photoproducts produced by UV radiation. 254 nm is known to be the most mutagenic wavelength in this context, but cyclobutan pyrimidin dimers and 6-4 photoproducs are also produced by UVB light. Whereas UVC light doesn't reach the earth's surface because it's absorbed by the ozonosphere and cannot penetrate the upper dermal layers, UVB is responsible for the formation of cyclobutan pyrimidin dimers and 6-4 photoproducs in human skin. Nevertheless we used UVC irradiation to damage the reporter constructs. To estimate NER capacity of cells using a host cell reactivation assay, it is necessary to provide a plasmid harboring a sufficient number of CPDs and 6-4 photoproducts. It doesn't matter if the CPDs and 6-4 photoproducts were generated by UVB or by UVC light. Certainly this conclusion applies only for the irradiation of plasmid DNA and not for the irradiation of whole skin or skin cell cultures where various side effects of the irradiation can be expected.

Using UVC light, a defined amount of UV damages (only CPDs and 6-4PPs) can be induced without running the risk of heat damages or provoking further damages, which can be generated by using broad band UV radiation. Therefore most of the studies in the field of host cell reactivation (addressing the NER pathway) were carried out using UVC light to damage the reporter plasmids.

The DsRed plasmid we used as reporter (a gift from the Natural and Medical Sciences Institute in the University of Tübingen (NMI)) was constructed by replacing the pEGFP-Cassette of pEGFP-N1 with the DsRedexpress cassette of DsRedexpress (Clonetech; Cat. No 632412). The plasmids were propagated in the *Escherichia coli *strain K12. Plasmid DNA isolation was performed using the Qiagen Plasmid Mega-Kit (Qiagen) according to the manufacture's protocols.

**Figure 2:**
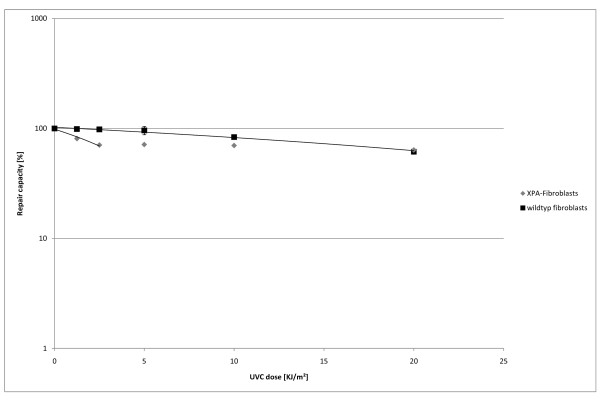
**Repair capacity of wildtype and XPA fibroblasts using different intensities for plasmid DNA irradiation**. Cells were transfected with a mixture of irradiated DsRedexpress and pEGP-N1 in molar ratio 3:1. DsRed plasmids were irradiated with 1.25, 2.5, 5.0, 10.0 and 20 kJ/m² UVC prior to transfection. Repair capacity was calculated according to the results of FACS 36 h post transfection. The experiment was carried out in triplicates.

To determine the irradiation dose required, we used XPA and wild-type fibroblasts. The UVC doses for plasmid damaging were chosen in dependence on the doses suggested by Roguev and Russev [3]. As figure 2 shows, the sequence encoding for the fluorophor remained mainly functional even after it was irradiated with 20 kJ/m^2^.

The number of cells expressing the plasmid irradiated with 20 kJ/m² is nearly the same in wild-type and XPA fibroblasts. This suggests that the intensive irradiation induces more damage than the cell is capable of repairing or that besides cyclobutan-pyrimidin-dimers or 6-4-photoproducts other damages are induced. The plasmid irradiated with 10 kJ/m² was repaired by 85% of the wild-type fibroblasts, but the plasmid irradiated with less than 5 kJ/m² was repaired by over 95% of the cells. In consideration of the standard deviation, we decided to use an irradiation dose of 5 kJ/m² UVC for all further experiments.

### Determination of the plasmid restoration

In addition to the irradiated plasmid, we used a second unirradiated control plasmid to correct any effects the transfection process might have on the repair capacity of cells. This also allows different experimental approaches to be compared.

**Figure 3:**
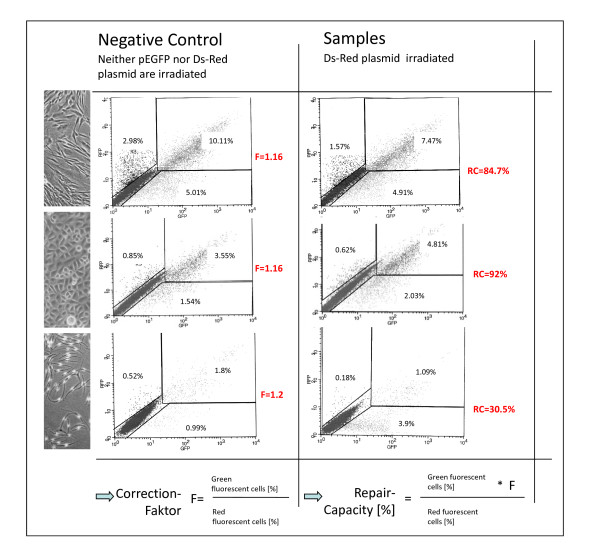
**Calculation of DNA repair capacity**. Primary fibroblasts, keratinocytes and melanocytes were transfected either with a mixture of irradiated DsRedexpress and pEGFP-N1 or with a mixture of unirrdiated DsRedexpress and pEGFP-N1. FACS analysis was carried out 36 h post transfection. The negative control serves as reference for data interpretation and to correct the results for potential variations in mixing ratio and point of time for FACS analysis.

Experiments using similar quantities of pEGFP and DsRedexpress for transfection showed that DsRed is underexpressed in comparison to pEGFP (data not shown). In earlier studies [17], we have shown that the optimum molecular ratio of pEGFP to DsRed is 1:3. In addition, the exact ratio was determined for every experiment and the repair capacity was corrected by multiplying with the calculated factor which varied normally between 0.8 and 1.2 (see figure 3).

**Figure 4:**
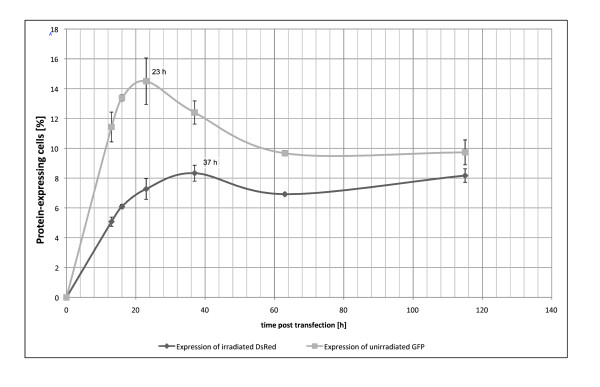
**Expression profile of irradiated DsRed and pEGFP**. Primary fibroblasts were cotransfected with irradiated DsRed and pEGFP-N1. Expression of DsRed and GFP was analyzed by FACS 13 h, 16 h, 23 h, 37 h, 63 h and 115 h post transfection. The experiment was carried out in triplicates.

**Figure 5:**
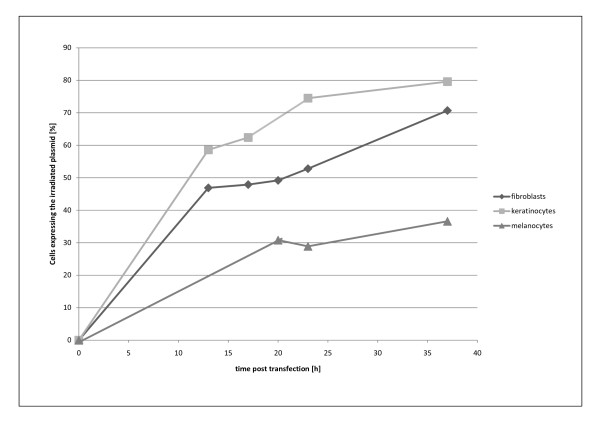
**Repair profiles of fibroblasts, keratinocytes and melanocytes during 36 h post transfection**. Primary fibroblasts, keratinocytes and melanocytes were cotransfected either with irradiated DsRed and pEGFP-N1 or unirradiated DsRed and pEGFP. Expression of both proteins was analyzed at different time points. The ratio of unirradiate DsRed to pEGP was used to normalize the data and to carry out data analysis. The experiment was carried out in triplicates. Day to day variation of the experiment all over the cell types is 3.5%.

To determine the ideal point in time for the FACS-analysis, the expression of both pEGFP and DsRed in cells cotransfected with both plasmids was observed in fibroblasts over a period of 115 h (figure 4). 23 h after transfection the highest expression of pEGFP was reached. The expression of pEGFP decreased up to 60 h post transfection and remained afterwards nearly unchanged up to 115 h post transfection. As described earlier [26] the peak of pEGFP expression occurs due to the fact that the maturation of DsRedexpress proceeds via GFP like intermediate. The highest expression of DsRed was reached 37 h after transfection. This is also true for keratinocytes and melanocytes (figure 5). Therefore, in the Cyclosporin A experiment the FACS-analysis was carried out 36 h after transfection.

### Cyclosporin A

**Figure 6:**
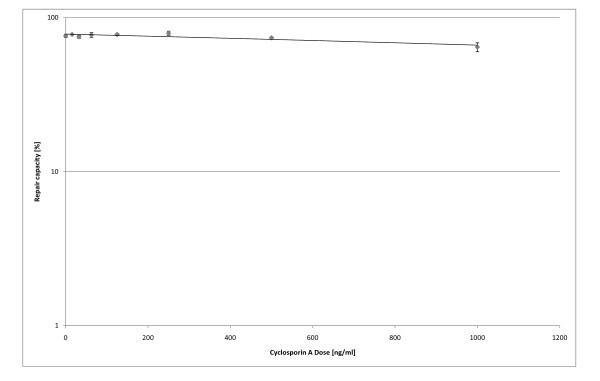
**Influence of Cyclosporin A on the DNA repair capacity of dermal fibroblasts: Dose-response relationship**. Fibroblasts were treated 24 h with different doses of Cyclosporin A prior to transfection. Cells were transfected with a mixture of irradiated DsRed (5 kJ UVC/m²) and pEGFP-N1.Post transfection, the cells were incubated for 36 h with Cyclosporin containing culture media. The experiment was carried out in triplicates.

**Figure 7:**
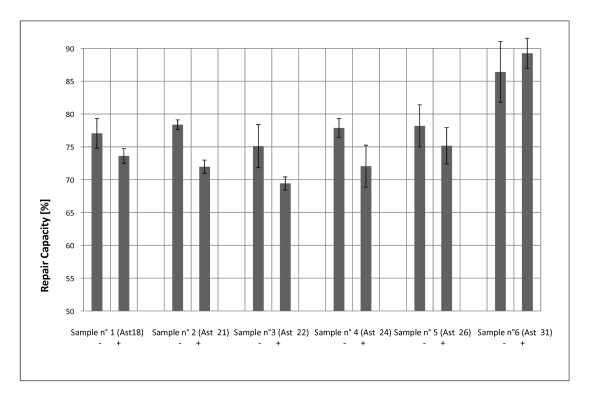
**Influence of Cyclosporin A on the DNA repair capacity of dermal fibroblasts: Influence of Cyclosporin A on the DNA repair capacity of different donors: **Further experiments using primary fibroblasts of 7 different donors were carried out using a Cyclosorin A dose of 1 μg/ml prior to and post transfection. FACS analysis took place 36 h post transfection. The experiments were carried out in triplicates (6b).

We finally validated the usefulness of this technique by determining the influence of Cyclosporine A on the DNA repair capacity of human dermal fibroblasts. The intake of Cyclosporin A is known to increase the risk of malignant skin changes [28]. Influences on the DNA repair capacity have already been shown for primary lymphocytes (via Host Cell Reactivation) [28] and keratinocytes (via direct analysis of the amount of CPDs) [29]. However, the direct analysis of DNA damages detects only the excision of the damages and does not detect the complete functional restoration of the damaged DNA sequence (DNA repair capacity). Using our modified assay, we have shown (figure 6 and figure 7) that Cyclosporin A leads to a dose-dependent decrease of the repair of UV-induced DNA damages in primary fibroblasts.

### Experimental design to compare the repair capacity of different skin cell types from one single donor

Based on the results above we designed an experimental setup to compare the repair capacity of different skin cell types from a single donor. After isolation, the cells were cultivated until the cell numbers were sufficient for experimental purposes. The different cells were plated into six well plates and transfected with FuGeneHD after they had been treated with either culture media containing the control or test substance. The FACS analysis was done 36 h after transfection.

The assay described here not only allows inter-individual variations in the DNA-repair capacities to be quantified but also those between the different cell types. For the first time, primary melanocytes and keratinocytes can be used in the Host Cell Reactivation Assay. These cells are considered to be the major target of UVB radiation as the vast majority of UVB is absorbed within the epidermis [16]. Enhancing the repair capacity of these cells via different substances could have a protective effect against the development of skin cancer as well as on premalignant skin lesions, particularly because epidermal cells are more easily accessible to (cosmetic) ingredients than dermal cells.

In comparison to the time-consuming electroporation procedure we have described earlier, liopofection saves time (because less working steps are needed) and only 5% of the plasmid DNA and 20% of the cells are needed.

## Conclusions

The efficient removal of DNA lesions is an important mechanism to prevent malignant transformation and tumor progression. In this context the Host Cell Reactivation Assay is a promising tool because it can serve to identify substances with positive effects on the repair capacity, which could be used as active ingredients in cosmetics. A further possible field of application is the confirmation/exclusion of negative influences on the repair capacity. Such undesired effects of cosmetic and drug ingredients could abet the development of malignancies. With respect to skin cells, cancer can appear in the general population, not only as a result of exposure to sunlight, but also due to a reduction in DNA repair, as seen in XP Patients.

In comparison with methods described earlier, this modified assay determines the repair capacity of different skin cell types to screen potential cosmetic ingredients. In addition, we describe a method to isolate all main skin cell types from one single biopsy. The method we developed is reliable and affordable. The only special equipment needed is a Fluorescent Activated Cell Sorter.

## Methods

### Reagents

10 × Trypsin-EDTA (PAA, Cat. # L11-003)

10 × Trypsin (PAA, Cat.# L11-001)

Collagenase D (Roche, Cat. # 11088866001)

Dispase II (Roche, Cat. # 04942078001)

Fetal Bovine Serum (PAA, Cat. # A15-251)

Gentamycin (PAA, Cat. # P11-005)

QiaFilter Plasmid Giga Kit (Qiagen, Cat. # 12291)

FuGene HD (Roche, Cat. # 04709705001)

Accutase (PAA, Cat.# L11-007)

FEI- Melanocyte Culture-Medium according to [18]

Keratinocyte Growth Media (Lonza, Cat. # CC-3111)

Fibroblast-Culture-Medium: DMEM High Glucose (PAA, Cat. # E15-843) containing 10% FBS and 0.5% Gentamycin (PAA, Cat, #P11-005)

### Equipment

CO_2_-Incubator (Heraeus Instruments)

Contrasting phase microscope (Carl Zeiss)

Laminar flow cabinet (Thermo Fisher)

Fluorescence Activated Cell Sorter FACSCalibur (BD Biosciences)

Curved tip forceps (Helbig)

Straight tip forceps (Helbig)

Petri dishes (Nunc)

Scalpel (BBraun)

Cell Strainer (BD Biosciences)

50 ml/15 ml reaction tubes (TPP)

2 ml reaction tubes (Sarstedt)

Nunclon Culture Flasks with vent/close cap (Nunc)

Nunclon 6-well Plates (Nunc)

### Isolation of fibroblasts, keratinocytes and melanocytes from skin biopsies

The biopsies are stored in PBS at 4°C until they are required. For the isolation of keratinocytes, it is important not to store the biopsies for longer than 24 h. If only melanocytes and fibroblasts are going to be isolated, the biopsies can be stored for up to 72 h.

The experiment was approved by the ethics committee of the Landesärztekammer Baden- Württemberg (Az 187-03). All donors were informed about the study and have given their consent.

A biopsy was transferred into a reaction tube filled with 20 ml of 70% isopropanol using sterile forceps. The tube was shaken vigorously. Afterwards, the biopsy was transferred into PBS using another pair of sterile forceps. After shaking the tube vigorously again, both wash steps were repeated.

#### Tissue preparation

The biopsy was transferred into a sterile petri dish using another pair of sterile forceps and placed upside down. Dermis was freed from fat and connective tissue and afterwards dissected into pieces of 3 × 3 mm. The pieces of tissue were incubated with 5 ml Dispase II for 4 h at 37°C or 14 h-16 h at 4°C. Afterwards epidermis and dermis were separated using fine sterile forceps.

#### Fibroblasts

**T**he dermal pieces were incubated with 3 ml Collagenase D for 2 h at 37°C and then transferred into a T_175 _culture flask. 10 ml Fibroblast Growth Medium were added.

#### Keratinocytes and Melanocytes

The epidermal sheets were incubated for 5 min at 37°C with 5 ml Trypsin (1 ×, diluted in PBS). Afterwards, the cells were separated by pipetting up and down for another 5 min. To stop the trypsin reaction, 5 ml PBS with 10% FBS were added. The cell suspension was passed through a cell strainer which was rinsed twice with PBS. Afterwards, the cells were collected by centrifugation (200 × g, 10 min). 2*10E6 cells were plated onto a T_75 _culture flask and either 10 ml Keratinocyte- or Melanocyte- Growth Medium were added.

### Cell culture

Cells are grown in a humidified CO_2_-Incubator (5% CO_2_) and the culture medium was changed 3 times a week. The cells were passaged when they reached 70% confluency. Therefore the culture-medium was aspirated, the culture was rinsed with PBS and either 5 ml 1 × Trypsin-EDTA (Melanocytes and Fibroblasts) or 10 ml Accutase (Kreatinocytes) per T_75_-flask were added. The flasks were incubated at 37°C until the cells start to disconnect from each other and from the culture flask. The cell displacement was encouraged by gentle tapping the sides of the flasks. When all cells were detached, the trypsin reaction was stopped by adding 10 ml of PBS with 10% FBS and the cells were collected by centrifugation (3 min, 500 × g). The cells were plated onto a T_175 _flask at a density from 2.5 to 5*10E5 cells.

### Plasmid-DNA-Isolation/UV-Irradiation

The pEGFP-n1 (Clonetech; Cat. No. 6085-1 GenBank: U55762.1) and DsRedexpress were propagated in *E. coli *K12 and purified as described in the CompactPrep Plasmid Mega/Giga Purification Handbook. UV irradiation of the DsRed plasmids was carried out in a cross linker (Stragene UV Stratalinker 1800) at 254 nm. Therefore small droplets (70 μl of Plasmid solution) were dispersed into a petri dish and irradiated with 5 kJ.

### Transfection

5*10E4 cells/well (Fibroblasts, Keratinocytes) and 1*10E5 cells/well (Melanocytes) were seeded in 6-well-plates and cultured until the monolayer was reached a confluency of 80-90%. To prepare transformation complex, the DNA was diluted with Ham's F12 to a concentration of 2 μg plasmid DNA-Mix/100 μl Ham's F12. 100 μl diluted DNA/transfection were mixed with 7 μl FuGene HD in a sterile polystytrene tube without allowing contact with the walls of the plastic tube and mixed gently by vortexing. The transfection reagent:DNA complex was incubated for 15 min at room temperature and added to the culture medium below the surface. Afterwards the plate was swirled to ensure distribution over the entire surface.

### FACS Analysis

Cultures were washed with PBS to rinse off the remaining culture medium. 500 μl 1 × Trypsin-EDTA (Melanocytes and Fibroblasts) or 500 μl Accutase (Kreatinocytes) per well were added and the plates were incubated at 37°C until the cells start to disconnect from each other and from the substrate. The dissociation was encouraged by gently tapping the sides of the plates and stopped after complete dissociation by adding 500 μl of PBS with 10% FBS. The cells were collected by centrifugation (3 min, 500 × g), resuspended in 100 μl PBS with 10% FBS and analyzed by FACS.

## Abbreviations

6-4 PP: 6-4-Photoproduct; CAT: Chloramphenicol Acetyl Transferase; CO_2_: Carbon Dioxide; CPD: Cyclobutan Pyrimidin Dimer; DNA: Desoxyribonucleic Acid; FACS: Fluorescent Activated Cell Sorter; FBS: Fetal Bovine Serum; GFP: Green Fluorescent Protein; HCRA: Host Cell Reactivation Assay; NER: Nucleotide Excision Repair; UV: Ultra Violet

## Competing interests

The authors declare that they have no competing interests.

## Authors' contributions

**BK **carried out parts of the cell isolation, the plasmid DNA-isolation and the irradiation, the transfection experiments, the FACS analysis and drafted the manuscript. **MK **carried out parts of the cell isolation experiments and helped to draft the manuscript. **KN **carried out parts of the cell isolation, helped in establishing the DNA-isolation and irradiation method and the FACS analysis. **GD **participated in the interpretation of data and revised the manuscript critically. **BJ **conceived of the study, and participated in its design and coordination and helped to draft the manuscript. All authors read and approved the final manuscript
